# Influence of Different Urban Structures on Metal Contamination in Two Metropolitan Cities

**DOI:** 10.1038/s41598-019-40180-x

**Published:** 2019-03-20

**Authors:** Badr H. Alharbi, Mohammad J. Pasha, Mohammed Ahmad S. Al-Shamsi

**Affiliations:** National Centre for Environmental Technology (NCET), Life Science & Environment Research Institute (LSERI), King Abdulaziz City for Science & Technology (KACST), Riyadh, Saudi Arabia

## Abstract

The influence of urban structures and land use patterns of metropolitan cities on the distribution of contaminants is not well understood. In this study, two metropolitan cities [Jeddah (a typical corridor city) and Madinah (a typical compact city)], featuring different spreading patterns and urban structures, were selected to investigate the contamination level and potential risk caused by metals (i.e., Pb, Zn, Co, Fe, Al, Cr, Cu, Ni, and Mn) associated with urban dust. The findings of this study show that a metropolitan city with a limited variety of activities and a polar center (e.g., Madinah) displays a typical distribution pattern of metals, i.e., concentrations of metals increase gradually toward the center of the city. In contrast, a metropolitan city with multiple major activities (e.g., Jeddah) displays a different distribution pattern, controlled by multiple key actors (e.g., seaports, oil refineries, and desalination/power plants) able to shift the location of highest contamination away from the city center. The above findings are supported by the results of several contamination and health indices. In Jeddah, the highest Pb contamination was found near an oil refinery based on geoaccumulation index (Igeo), contamination factor (CF), enrichment factor (EF), and ecological risk (E_i_) values; whereas, the highest Zn contamination was found near a seaport, based on EF, CF, and E_i_ values. However, in Madinah, the contamination indices indicate that the most contaminated locations are near the city center. The highest non-carcinogenic health risk in Jeddah was found near an oil refinery and in the city center; whereas in Madinah, it was found mainly in the city center. Although there is no significant risk of cancer due to metals associated with dust in the two cities, Cr, representing a health risk contribution of >24%, was the major contributor of non-carcinogenic health risk in the two metropolitan cities.

## Introduction

The risk of human exposure to metals threatens public health, as they can cause multiple adverse health effects^1,2^. The toxicity of metals is recognized as caused by the potential production of highly reactive species, such as reactive oxygen species (ROS) and other free radicals, causing several effects on cell structure including DNA damage, protein depletion, and lipid peroxidation^1^.

Although some metals are essential for human nutrition, such as copper (Cu), zinc (Zn), and chromium (Cr), an excess amount of them causes several health and environmental risks^3^. For instance, Cr is categorized by the International Agency for Research on Cancer (IARC) as a human carcinogen (Group 1 category) because of its potential mutagenic properties. In addition, the biological processes of plants are heavily affected by Cr toxicity^4^.

Some metals, such as lead (Pb) and aluminum (Al), are nonessential, with no known beneficial effects for living organisms^3^. However, Pb is classified by the IARC as possibly carcinogenic to humans (Group B2 category), and chronic exposure to Pb causes infertility, paralysis, mental retardation, brain and kidney damage, and other health problems^1,4^. Al is known to cause many diseases to humans including Alzheimer’s^1^. Therefore, metals are considered significant pollutants in the environment and living organisms.

Metals are generated from a wide range of anthropogenic and natural sources, including weathering of the Earth’s crust, industrialization, and mining^2^; however, in urban areas, heavy traffic is considered a major source of metals^5^. Metals associated with dust are transferred away from the generation sources via wind and settle in urban areas^6,7^.

Thus, the exposure risk of metals in different metropolitan cities has become a major concern of public health practitioners. Each city has a unique urban structure and land use pattern, which cause different exposure levels of contaminants to its inhabitants. The arrangement and relationships of the arrays of services, goods, and residents generate a unique urban structure for every metropolitan city, which is continually changing over time^8,9^. The influence of the urban structure of cities and distribution patterns of land uses on the environment is not well understood^10^. Borrego, *et al*.^10^ suggested three main distribution patterns of metropolitan cities based on different land use patterns: disperse city, corridor city, and compact city.

A great deal of effort has been put forth to assess the contamination levels and health risk exposures caused by metals associated with urban dust in multiple cities worldwide, including Huludao, China^11^; Maha Sarakham, Thailand^12^; Beijing, China^13^; Guiyang, China^14^; Tianjin, China^15^; Nanjing, China^16^; Bayan Obo Mining Region, China^17^; Atakumosa, Nigeria^18^; Kagiso, South Africa^19^; Gyumri, Armenia^20^; Shiraz, Iran^21^; Sialkot, Pakistan^22^; Zhuzhou, China^23^; Baoton, China^24^; Jeddah, Saudi Arabia^25^; and Riyadh, Saudi Arabia^26^.

These studies focused on the contamination and health risk assessment for a single city, missing the opportunity to compare the distribution patterns of contamination and associated risks for different urban structures to highlight the similarities and differences that could lead to a common valuable conclusion and shared circumstances. In this study, the aim was to understand if urban structure shape and land use distribution affect the distribution patterns of metal contamination and risk hotspots in two metropolitan cities with different urban structures.

## Materials and Methods

### Description of Selected Sites

Two metropolitan cities with over 1 million inhabitants were selected carefully to represent two different urban structures. Jeddah was chosen as a typical city with a corridor urban structure, where the main activities of the inhabitants stretch along the shore of the Red Sea. Madinah was chosen as a typical compact city with central/polar activity in the city center, where an ancient mosque drives the activities of daily life around it.

#### Jeddah

Jeddah is the second largest city in Saudi Arabia and the major seaport of the country and has an urban area of 1,765 km^2^ and a population of ~3.98 million^27^, with a 3.5% annual growth rate. This city is located on the eastern shore of the Red Sea at a latitude of 29.21°N and a longitude of 39.7°E. Northwest winds are the prevailing winds of Jeddah. During the summer, the city faces high temperature and humidity^28^. Jeddah consists of several mobile and stationary sources of emissions, including 1.4 million vehicles, an oil refinery, a desalination plant, a power generation plant, and multiple industrial areas^29^.

#### Madinah

Madinah currently is a major city in Saudi Arabia and historically was the capital city of the Islamic Empire in the 7^th^ century AD with multiple ancient locations that attracted visitors from all over the world. Geographically, the city is located at a latitude of 24.28°N and a longitude of 39.7°E, and it has a population of 1.3 million^27,30^. The average altitude of Madinah is 620 m above sea level, and it has an urban area of approximately 293 km^2^.

### Sampling and preparation

**Figure 1:**
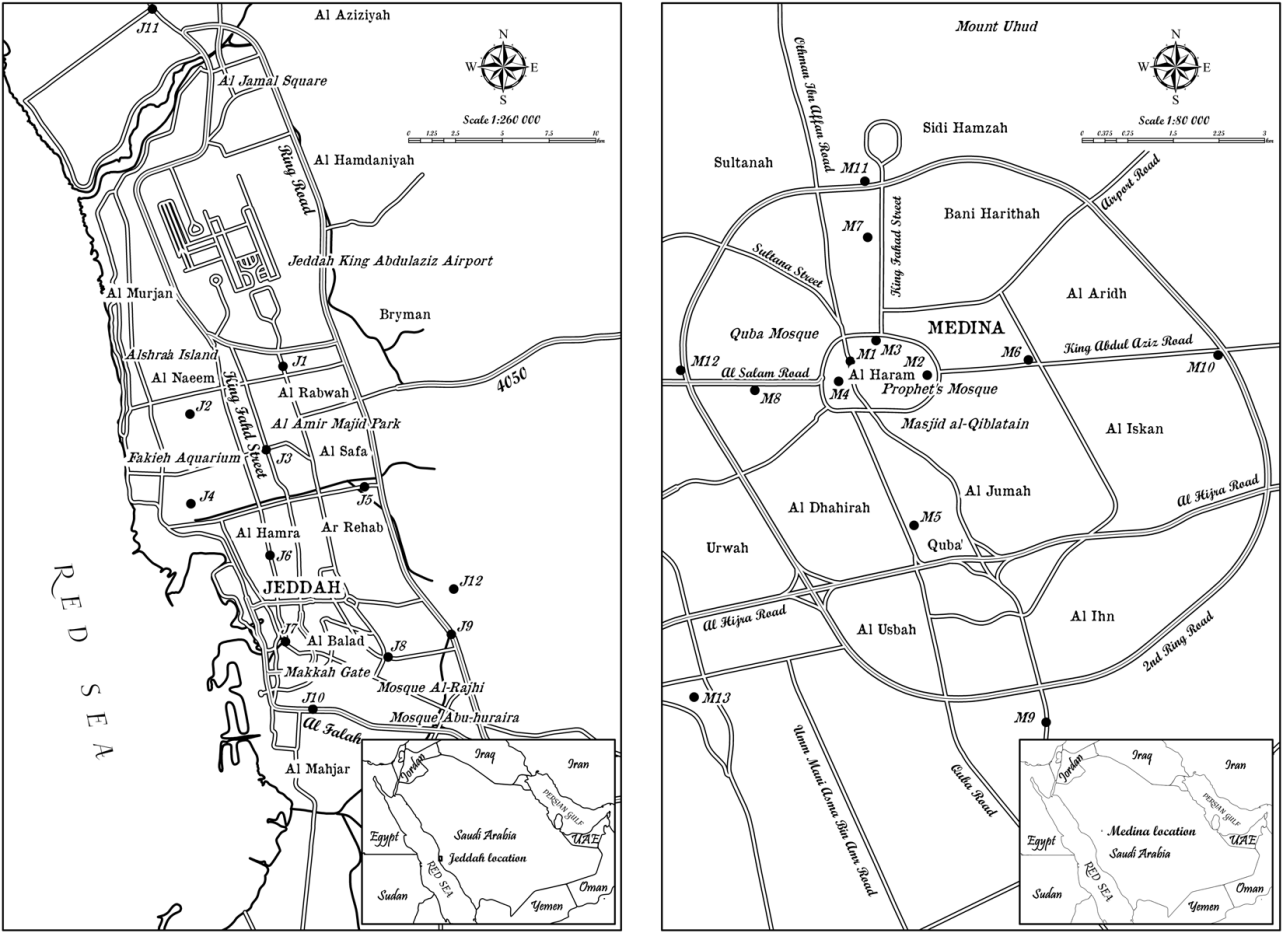
Map of the sampling locations at the two metropolitan cities in Saudi Arabia; Jeddah is on the left side, Madinah is on the right side.

Randomly, twenty-five sampling sites were selected to represent the two metropolitan cities (Madinah and Jeddah) as shown in Fig. 1. At each site, a composite sample of dust was collected during the summer of 2014 using brushes (1-inch head width, ACE) through sweeping from several spots within the location of each selected site. Approximately 100 g of the dust was collected in a plastic bag (200 g; Sunpet Co.) from each site location, delivered in portable travel coolers to the laboratories within 3 days of collection. In the laboratory, samples were dried and sieved (Laboratory test sieve: 0.75 µm, Endecotts, Ltd.). It is worth to be mentioned that the sampling location of M8 was collected from a location near a construction site at the early stage of excavation, assuming that the collected dust was accumulated recently from the generated dust in few meters in-depth of the geological site.

### Materials and chemical analysis

For laboratory analyses, the following chemicals were purchased: hydrofluoric acid 40% (BDH, UK), hydrochloric acid 37% (Merck, Germany), nitric acid 65% (Emsure, Germany), hydrogen peroxide 35 wt% solution (Acros, USA).

The analysis of metals associated with urban dust samples were performed as described by Clesceri, *et al*.^31^ using a microwave digestion system (MDS-2100, CEM). First, a microwave Teflon tube was filled with 300 mg of dust sample, followed by adding 10 ml of Milli-Q water (18.2 MQ-CM, PURELAB Option). The following chemicals were added in a sequence to the Teflon tube: 5 ml of nitric acid, 2 ml of hydrofluoric acid, 1 ml of hydrochloric acid, and few drops of hydrogen peroxide. According to the method described by US.EPA^32^, the Teflon tubes were placed in the microwave for 30 min and at a pressure of 120 psi. Inductively coupled plasma mass spectrometry (ICP-MS, Agilent 7900) was used to analyze the digested samples of urban dust. ICP Multi Elemental Standard Solution VI (CertiPUR®, Merck Co.) was used for calibrations of the selected metals.

### Contamination indices

#### Enrichment factor (EF)

EF was calculated using the following equation as provided by Taylor and McLennan^33^:

1 $${\rm{EF}}={({\rm{C}}}_{{\rm{n}}}{/{\rm{C}}}_{{\rm{ref}}})/{({\rm{B}}}_{{\rm{n}}}{/{\rm{B}}}_{{\rm{ref}}}),$$

where C_n_ is the concentration of the examined metal, C_ref_ is the concentration of the reference metal, B_n_ is the background value of the examined metal, and B_ref_ is the background value of the reference metal in Earth’s crust. The reference metal in this study was aluminum.

Five categories were proposed by Sutherland^34^ to describe the contamination level based on of the value of EF as follows: EF < 2, depletion to minimal contamination; 2 ≤ EF < 5, moderate contamination; 5 ≤ EF < 20, significant contamination; 20 ≤ EF < 40, very high contamination; and EF > 40, extremely high contamination.

#### Contamination factor (CF)

CF is the single-element index and can be calculated by the following equation as indicated by Rastmanesh, *et al*.^35^:

2 $${{\rm{C}}}^{{\rm{i}}}{\rm{f}}={{{\rm{C}}}^{{\rm{i}}}}_{0-1}/{{{\rm{C}}}^{{\rm{i}}}}_{{\rm{n}}},$$

where C^i^f is the contamination factor for the metal of interest, C^i^_0-1_ is the concentration of the metal in the sample, and C^i^_n_ is the background concentration^33^. Four CF-categories are used to classify the contamination level of metals in the sampling locations as follows: CF < 1, low contamination factor; 1 ≤ CF < 3, moderate contamination factor; 3 ≤ CF < 6, considerable contamination factor; and CF ≥ 6, very high contamination factor.

#### Pollution load index (PLI)

PLI was calculated based on the collection of the CF values of all metals in each sampling location, as described by Tomlinson, *et al*.^36^ in the following equation:

3 $${\rm{PLI}}={}^{{\rm{n}}}\surd ({\rm{CF1}}\,\ast \,{\rm{CF2}}\,\ast \,{\rm{CF3}}\,\ast \,{\rm{\ldots }}{\rm{\ldots }}{\rm{.}}\,{\rm{CF}}\,{\rm{n}}),$$

where *n* is the number of metals studied and *CF* is the contamination factor calculated as in Equation (2). Three categories of PLI are presented as follows: PLI < 1, perfection; PLI = 1, presenting baseline levels of pollutants; and PLI > 1, deterioration of site quality. Due to the abundance of aluminum and iron in earth’s crust, they were excluded from calculating the PLI values of the sampling locations.

#### Geo-accumulation index (Igeo)

Igeo was calculated as indicated by Muller^37^ using the following equation:

4 $${\rm{Igeo}}={\mathrm{log}}_{{\rm{2}}}(\mathrm{Cn}/{\rm{1}}{\rm{.}}{\rm{5}}\,\mathrm{Bn}),$$

where Cn is the measured concentration of the metal in sample and Bn is the background concentration of the metal. The seven categories of the geo-accumulation index indicated by Muller^37^, Huu^38^ are presented as follows: Igeo < 0, uncontaminated (Igeo-class 0); 0 ≤ Igeo < 1, uncontaminated to moderately contaminated (Igeo-class 1); 1 ≤ Igeo < 2, moderately contaminated (Igeo-class 2); 2 ≤ Igeo < 3, moderately to strongly contaminated (Igeo-class 3); 3 ≤ Igeo < 4, strongly contaminated (Igeo-class 4); 4 ≤ Igeo < 5, strongly to extremely contaminated (Igeo-class 5); and Igeo > 5, extremely contaminated (Igeo-class 6).

#### Nemerow pollution index (NPI)

NPI was proposed by Nemerow and Sumitomo^39^ as an overall quality expression tool of pollution, and it has been used later in many applications including the pollution of metals in several media^40,41^. NPI was calculated using the following equation:

5 $${NPI}=\frac{\sqrt{{P}{{I}}_{{Max}}^{2}+\,{P}{{I}}_{{Average}}^{2}}}{2}$$

6 $${P}{{I}}_{{i}}=\,\frac{{{C}}_{{i}}}{{{S}}_{{i}}}$$

where PI_i_ is the single factor pollution index, C_i_ is the content of pollutant, S_i_ is the natural background content of the pollutant, PI_max_ is the maximum value of the pollutants, and PI_average_ is the average value of the pollutants. Four categories of NPI are given by^42^ to classify the level of pollution as follows: *P* ≤ 1, un-contaminated; 1 < *P* ≤ 2, slightly contaminated; 2 < *P* ≤ 3, moderately contaminated; and *P* > 3, severely contaminated. For the single pollution index (PI_i_) that has been used to evaluate the site pollution by each single metal^41^, five categories were used as follows: PI_i_ ≤ 1, safety; 1 < PI_i_ ≤ 2, slight pollution; 2 < PI_i_ ≤ 3, mild pollution; 3 < PI_i_ ≤ 5, moderate pollution; and PI_i_ > 5, severe pollution.

#### Degree of similarity

The degree of similarity or discrepancy of the metals among the different sites was calculated using the following divergence ratio (CD)^43^.

7 $$C{D}_{jk}=\sqrt{\frac{1}{p}\sum _{i=1}^{p}\,{(\frac{xij-xik}{xij+xik})}^{2}}$$

Here, *x*_*ij*_ is the average concentration of metal *i* at a certain site, *j* and *k* are two sampling sites, and *p* is the number of metals. If the calculated CD tends toward zero, measurements from both sites are considered similar, whereas if the CD is closer to one, measurements from the two sites are considered different.

### Risk assessment analysis

#### Ecological risk assessment

The risk index (RI), which was proposed by Hakanson^44^, was calculated via equation 8 to estimate the potential risk of metals associated with urban dust. The categories of RI, as indicated by Jiang, *et al*.^45^, Yi, *et al*.^46^, are shown in Table 1. Al and Fe were excluded when estimating RI because of their abundance in the Earth’s crust.

8 $${\rm{RI}}=\sum _{{\rm{i}}={\rm{1}}}^{{\rm{n}}}\,{{\rm{E}}}_{{\rm{i}}}=\sum _{{\rm{i}}={\rm{1}}}^{n}\,{{\rm{T}}}_{{\rm{i}}}{{\rm{C}}}_{{\rm{f}}}^{{\rm{i}}}=\sum _{{\rm{i}}={\rm{1}}}^{n}\,{{\rm{T}}}_{{\rm{i}}}\frac{{{\rm{C}}}_{{\rm{s}}}^{{\rm{i}}}}{{{\rm{C}}}_{{\rm{r}}}^{{\rm{i}}}}$$

where E_i_ is the toxic risk factor of metal i, i is a single metal, T_i_ is the toxic response factor of metal i [T_i_ values of the metals are Co = Cu = Ni = Pb = 5, Cr = 2, and Zn = Mn = 1]^44,47^, ${{\rm{C}}}_{{\rm{s}}}^{{\rm{i}}}$ is the measured concentration of metal i in the sample, ${{\rm{C}}}_{{\rm{f}}}^{{\rm{i}}}$ is the contamination factor of metal i, and ${{\rm{C}}}_{{\rm{r}}}^{{\rm{i}}}$ is the reference value of metal i (the value of metal i in the Earth’s crust).

**Table 1:** Grading standards used to evaluate the ecological risk factor (Ei) and the ecological risk index (RI).

Ei	Potential ecological risk for single metal	RI	Ecological risk for all metals
Ei < 5	Low	RI < 25	Low
5 ≤ Ei < 10	Moderate	25 ≤ RI < 50	Moderate
10 ≤ Ei < 20	Considerable	50 ≤ RI < 100	Considerable
20 ≤ Ei < 40	High	RI ≥ 100	High
Ei ≥ 40	Very high		

#### Health risk assessment

Cancer Risk: The risk of the carcinogenic metals (i.e., Co, Ni, and Cr) was calculated using the following equations:

9 $${\rm{C}}{\rm{a}}{\rm{n}}{\rm{c}}{\rm{e}}{\rm{r}}\,{\rm{r}}{\rm{i}}{\rm{s}}{\rm{k}}={\rm{L}}{\rm{A}}{\rm{D}}{\rm{D}}\times {\rm{S}}{\rm{F}}$$

10 $${\rm{LADD}}=\frac{{\rm{C}}\times {\rm{EF}}}{{\rm{PEF}}\times {\rm{AT}}}\times (\frac{{{\rm{R}}}_{{\rm{inh}}{\rm{child}}}\times {{\rm{ED}}}_{{\rm{child}}}}{{{\rm{BW}}}_{{\rm{child}}}}+\frac{{{\rm{R}}}_{{\rm{inh}}{\rm{adult}}}\times {{\rm{ED}}}_{{\rm{adult}}}}{{{\rm{BW}}}_{{\rm{adult}}}})$$

where SF is the slope factor (kg day/mg). As the SF values for Co, Ni, and Cr, 9.8, 420, and 0.84 kg day/mg were employed respectively^48,49^. LADD is the lifetime average daily dose (mg /kg/day), C is the contaminant exposure-point concentration (mg/m^3^), EF is the frequency of exposure (180 day/year)^11,48^, PEF is the emission factor of particles (1.36 × 10^9^), ED is the duration of exposure (6 years for children and 24 years for adults)^50^, AT is the average time (72 × 365 days for carcinogens)^51^, R_inh_ is the rate of inhalation for children (7.6 m^3^/day) and adults (20 m^3^/day)^52^, and BW is the average body weight for children (16.8 kg) and adults (61.8 kg)^51^.

Rodricks, *et al*.^53^ indicated that a cancer risk value greater than or equal to 1 in 1,000 is considered a significant risk, whereas a value lower than 1 in 1,000,000 is considered a negligible risk.

Non-cancer RiskThe risk for the non-carcinogenic metals (i.e., Al, Cu, Pb, Zn, Fe, and Mn) was calculated using the following equations:

11 $$\begin{array}{ll} & {\rm{N}}{\bf{o}}{\rm{n}} \mbox{-} {\rm{c}}{\rm{a}}{\rm{r}}{\rm{c}}{\rm{i}}{\rm{n}}{\rm{o}}{\rm{g}}{\rm{e}}{\rm{n}}{\rm{i}}{\rm{c}}\,{\rm{r}}{\rm{i}}{\rm{s}}{\rm{k}}({\rm{H}}{\rm{I}})={\rm{H}}{{\rm{Q}}}_{{\rm{i}}{\rm{n}}{\rm{g}}}+{\rm{H}}{{\rm{Q}}}_{{\rm{d}}{\rm{e}}{\rm{r}}{\rm{m}}{\rm{a}}{\rm{l}}}+{\rm{H}}{{\rm{Q}}}_{{\rm{i}}{\rm{n}}{\rm{h}}}\\ = & (\frac{{{\rm{D}}}_{{\rm{ing}}}}{{{\rm{RfD}}}_{{\rm{ing}}}})+(\frac{{{\rm{D}}}_{{\rm{dermal}}}}{{{\rm{RfD}}}_{{\rm{dermal}}}})+(\frac{{{\rm{D}}}_{{\rm{inh}}}}{{{\rm{RfD}}}_{{\rm{inh}}}})\end{array}$$

12 $${{\rm{D}}}_{{\rm{ing}}}=C\times \frac{{{\rm{R}}}_{{\rm{ing}}}\times \mathrm{EF}\times \mathrm{ED}}{\mathrm{BW}\times \mathrm{AT}}{\times \mathrm{10}}^{-{\rm{6}}}$$

13 $${{\rm{D}}}_{{\rm{dermal}}}=C\times \frac{\mathrm{ESA}\times \mathrm{SAF}\times \mathrm{DAF}\times \mathrm{EF}\times \mathrm{ED}}{\mathrm{BW}\times \mathrm{AT}}{\times \mathrm{10}}^{-{\rm{6}}}$$

14 $${{\rm{D}}}_{{\rm{inh}}}=C\times \frac{{{\rm{R}}}_{{\rm{inh}}}\times \mathrm{EF}\times \mathrm{ED}}{\mathrm{PEF}\times \mathrm{BW}\times \mathrm{AT}}$$

where RfD is the reference dose for the daily intake level of a particular contaminant, SAF is the skin adherence factor for children (0.2 mg cm^2^/day) and adults (0.07 mg cm^2^/day)^11,50^, ESA is the exposed skin area for children (2,800 cm^2^) and adults (5,700 cm^2^)^50^, DAF is the dermal absorption factor for all metals (unitless value of 0.001)^48,54^, and AT is equal to ED × 365 days for noncarcinogens^51^.

The hazard quotients of ingestion, inhalation, and dermal routes are HQ_ing_, HQ_inh_, and HQ_dermal_, respectively; the doses of urban dust (mg/kg/day) through ingestion, inhalation, and dermal exposure routes are D_ing_, D_inh_, and D_dermal_, respectively; the reference doses (mg/kg/day) of metals through ingestion, inhalation, and dermal exposure routes are RfD_ing_, RfD_inh_, and RfD_dermal_, respectively; and R_ing_ is the rate of ingestion for children (200 mg/day) and adults (100 mg/day)^50^.

The toxicity values of Cu, Pb, Zn, and Fe are unavailable for the inhalation pathway. Thus, the values of oral RfD were applied, assuming that metal absorption after inhalation would have impacts close to those of absorption through ingestion^52^. An HI less than 1 indicates no significant risk of non-carcinogenic effects, whereas an HI greater than 1 indicates that non-carcinogenic effects may occur^55^.

## Results and Discussion

The investigated contamination indices feature different levels and sensitivity toward contamination. Thus, inconsistencies in the indicated contamination levels can be anticipated^56^.

### Metal distribution patterns

**Figure 2:**
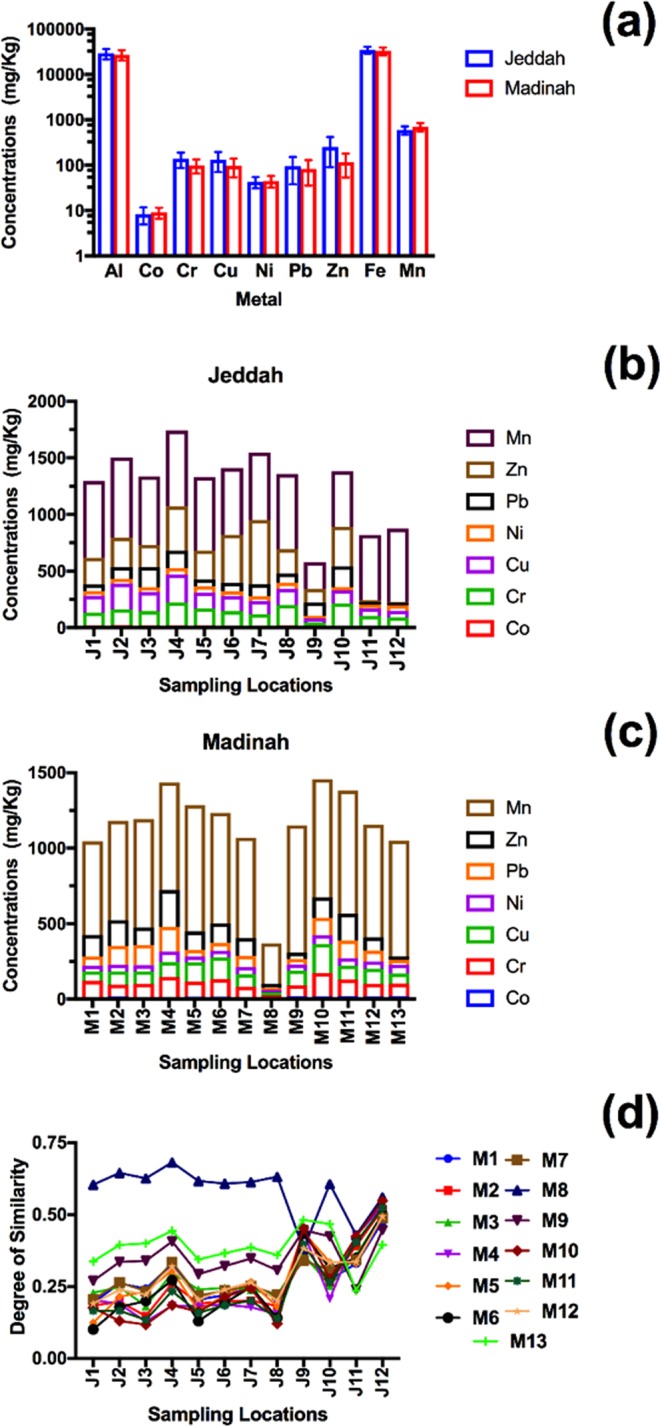
(**a**) the average metal concentrations in the two metropolitan cities in Saudi Arabia, error bars represent the standard deviations. (**b**,**c**) are the metal distributions in each sampling locations in Jeddah and Madinah, respectively. (**d**) is the degree of similarity and divergence of the two metropolitan cities.

The average concentrations of six metals (Al, Cr, Cu, Pb, Zn, and Fe) in all the sampling locations of Jeddah were higher compared to those of Madinah, as shown in Fig. 2(a). For instance, the average concentration of Zn in the sampling locations of Jeddah (254 ± 163 mg/kg) was 121% higher than that of Madinah (115 ± 63 mg/kg). For Cr, Cu, and Pb, the average concentrations in the sampling locations of Jeddah were 41%, 33%, and 14% higher, respectively, than those of Madinah.

The patterns of metal distribution in the two cities are shown in Fig. 2(b,c). Regarding the distribution pattern of Pb in Madinah, the highest concentrations were found in the city center in the sampling locations M4 (167 mg/kg), M3 (135 mg/kg), and M2 (125 mg/kg). The lowest concentrations of Pb in Madinah were found in the locations on the outer side of the ring road, such as M9 (38 mg/kg) and M13 (35 mg/kg) (the case of M8 was explained in the methodology section).

Concerning the distribution pattern of Pb in Jeddah, the highest concentration was found in the sampling location of J10 (182 mg/kg), which is the sampling location closest to an oil refinery in the city. Krastinyte, *et al*.^57^ found that Pb and Cr were the metals with the highest concentrations in the samples (dust-snow) collected near an oil refinery in Lithuania. The findings of the current study are in agreement with those findings.

Furthermore, high concentrations of Pb were found in the sampling locations J3 (176 mg/kg), J4 (155 mg/kg), and J9 (122 mg/kg). This could be related to activities of the city, such as power plant, desalination plant, industrial area and other major activities in the city.

Comparing the Pb distribution patterns of the two metropolitan cities, the lowest concentrations were found in the areas far from the city center, such as the sampling locations M9 and M13 in Madinah, whereas the highest were found in the city center (i.e., M2, M3, and M4). The high concentrations of Pb, Zn, and Cr in Madinah were clearly related to the compact pattern of the city, as the main activities are located in the city center. For Jeddah, the highest concentrations of Pb were in the city center (i.e., J3 and J4) as well as in areas near the main activities (i.e., J10 and J9), which were distributed along the corridor pattern of the city.

Madinah’s distribution pattern of Zn was similar to that of Pb, where the highest concentrations were found in M2 and M3 and the lowest concentrations in M8, M9, and M13. However, Jeddah’s distribution pattern of Zn was different from that of Pb, where the highest concentrations of Zn were found in the sampling locations J7 (567 mg/kg) and J6 (425 mg/kg), which are located near the seaport of the city and the shore of the Red Sea. Jahan and Strezov^58^ found very high concentrations of Zn in the water of five seaports in Australia, which exceeded the guideline acceptance levels. This suggests that human activities near seaports might cause Zn contamination in the surrounding areas. Comparing the Zn distribution patterns of the two metropolitan cities, they are relatively similar to those of Pb, with greater emphasis on the clear difference between the two urban patterns.

The pattern distribution of Cr in Jeddah was similar to that of Pb, where the highest values were near the city center (J4: 214 mg/kg) and the sampling locations near an oil refinery (J10: 212 mg/kg). For the pattern distribution of Cr in Madinah, it was relatively similar to that of Pb.

For Cu, Co, Ni, Al, Fe, and Mn, no clear distribution patterns could be identified nor the sources related to major activities in the two metropolitan cities.

### Similarity degrees between the two cities

The similarity matrix between the sampling locations within the city of Madinah was investigated (Supplementary Table S1). Generally, a degree of similarity below a value of 0.25 was present between sampling locations within the city of Madinah, except for three sampling locations (M8, M9, and M13). These three sampling locations have similarity values mostly between 0.25 and 0.6, which makes them dissimilar from the rest of the similarity matrix pattern of the city. These sampling locations seemed to be affected by a similar common site contamination source(s) of metals, which are concentrated in the city center. The sampling locations of M9 and M13, which are located away from the city center, have a degree of similarity of 0.163, which might indicate a common source(s) of metals; whereas, M8 is the most dissimilar from these two sampling locations and the rest of the sampling locations in the city. The status of M8 was described in methodology section.

The similarity pattern between the sampling locations within the city of Jeddah is presented in Supplementary Table S1. The similarity degree between sampling locations of the city is below 0.2, except for four sampling locations. Such as for Madinah, this similarity matrix might indicate a common shared source of metal contamination, presumably traffic. However, the similarity degree of the sampling locations J9, J11, and J12 has values ranging between 0.35 and 0.61, which might indicate a similar source of metals. The degree of similarity of the sampling location J10 with most of the other sampling locations is between 0.2 and 0.3, which might indicate a source of metals that is different from the other two previous patterns, which were for areas near the shipping roads of the seaport.

The comparison of the similarity matrices of the two metropolitan cities is shown in Fig. 2(d). The most dissimilar sampling locations between the two cities were found to be M8, M9, M13, J9, J11, and J12. Most of their similarity degree ranges from 0.33 to 0.68, indicating a different potential source(s) of metals compared to the rest of the sampling locations in the two metropolitan cities. M9 and M13 follow exactly the same similarity pattern as that of the other sampling locations in Jeddah. They had among the highest values of similarity degree with the sampling locations from J1 to J10, whereas the lowest values of similarity degree were for J11 and J12.

In contrast, J9, J11, and J12 showed the most dissimilarity with the sampling locations of Jeddah. However, M8, M9, and M13 were relatively dissimilar with J9, J11, and J12, which might indicate a variety of metal sources.

### Contamination indices of a single metal

#### Enrichment factor (EF)

**Figure 3:**
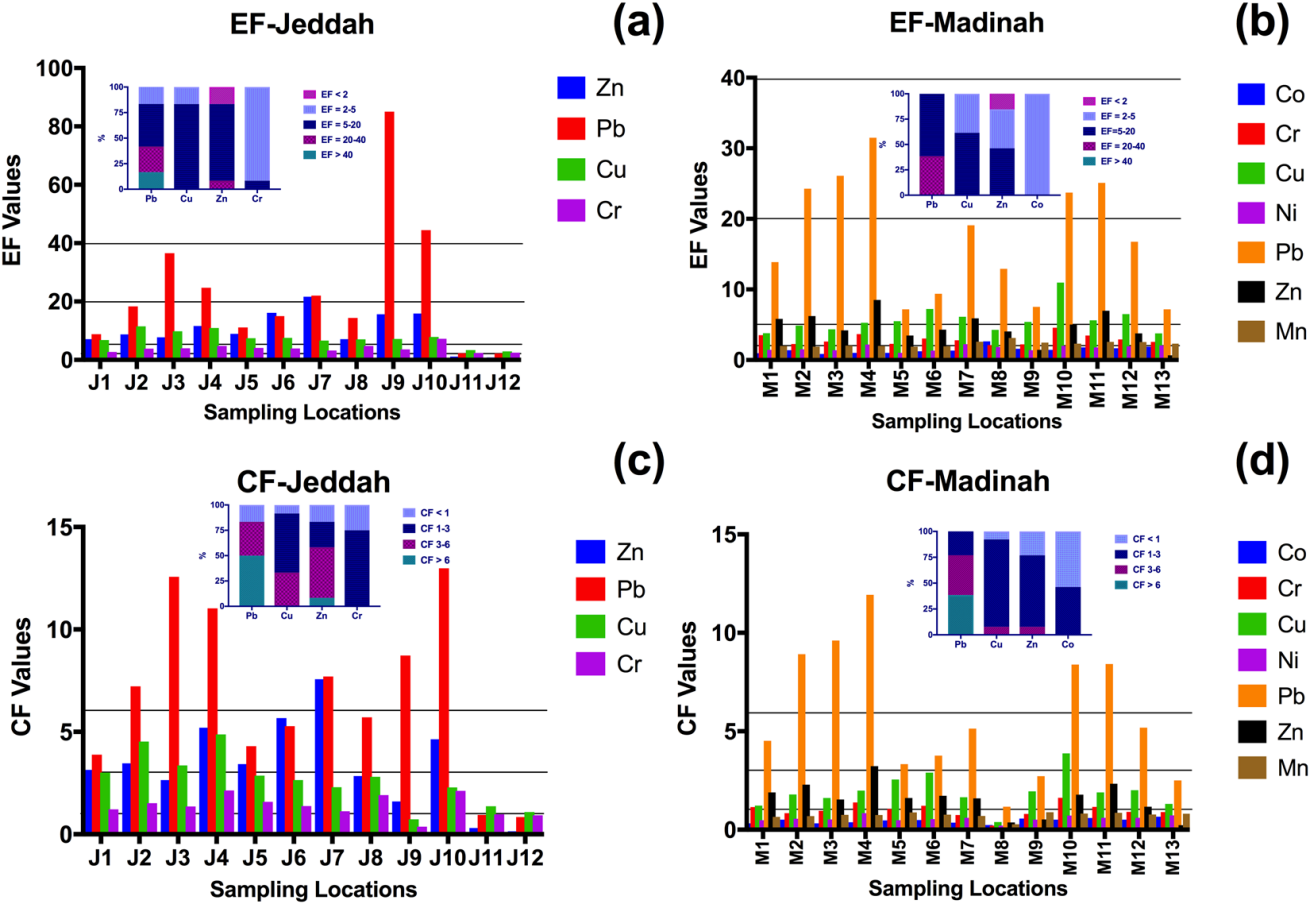
The enrichment factor (EF) values of Jeddah (**b**) and Madinah **(b**) and the contamination factor (CF) values of Jeddah (**c**) and Madinah (**d**) of the highest metals associated with urban dust. The small graphs inside the figure represent the percentage distribution of the CF and EF values.

The highest calculated EF values of the sampling locations in the two metropolitan cities are presented in Fig. 3(a) for Jeddah and (b) for Madinah (more data are displayed in Supplementary Table S2). The highest EF values for Pb and Zn were clearly related to the urban pattern of each city.

Extremely high contamination by Pb (EF > 40) was observed in Jeddah in the sampling locations near an industrial area (i.e., J9) and near an oil refinery (i.e., J10), which represent 16.6% of the study area, as shown in Fig. 3(a).

Very high contamination (40 > EF > 20) with Pb was observed for three sampling locations in Jeddah (J3, J4, and J7), and significant contamination (20 > EF > 5) was observed in the sampling locations J2, J6, and J8.

In Madinah, 38.5% of the sampling locations (i.e., M2, M3, M4, M10, and M11) were classified as very highly contaminated and 53.9% as significantly contaminated (20 > EF > 5) with Pb.

For Zn, only one sampling location in the two metropolitan cities was classified as very highly contaminated (40 > EF > 20), the one near the seaport of Jeddah (J7). Significant contamination (20 > EF > 5) with Zn was observed in 75% of the sampling locations in Jeddah compared to 38.5% in Madinah.

For Cu, 83.3% of the sampling locations in Jeddah were classified as significantly contaminated (20 > EF > 5) compared to 61.5% in Madinah.

For Cr, one sampling location in the two cities was classified as significantly contaminated (20 > EF > 5), namely the one near an oil refinery in Jeddah.

#### Contamination factor (CF)

The calculated CF values of the highest metals in the sampling locations of the two metropolitan cities are displayed in Fig. 3(c,d). The distribution patterns of the high CF values of Pb and Zn were affected by the urban structure of each city. However, the two cities, Jeddah and Madinah, were very highly contaminated (CF > 6) with Pb, with 50% and 38.5% of the sampling locations in Jeddah and Madinah contaminated, respectively. The most contaminated locations based on the CF index were found to be near the oil refinery in Jeddah (i.e., J10) and in the city center of Madinah (i.e., M3 and M4).

As with the EF index, the only location with very high Zn contamination (CF > 6) was near the seaport of the city of Jeddah (J7). Considerable contamination by Zn (6 > CF > 3) was observed in 50% of the sampling locations in Jeddah compared to only in one sampling location in Madinah, in the city center (M4).

For Cu, 33% of the sampling locations were considerably contaminated (6 > CF > 3) compared to only one sampling location in Madinah (M10). The rest of the investigated metals in the two cities were classified as causing low and moderate contamination (3 > CF > 1).

#### Geoaccumulation index (Igeo)

**Figure 4:**
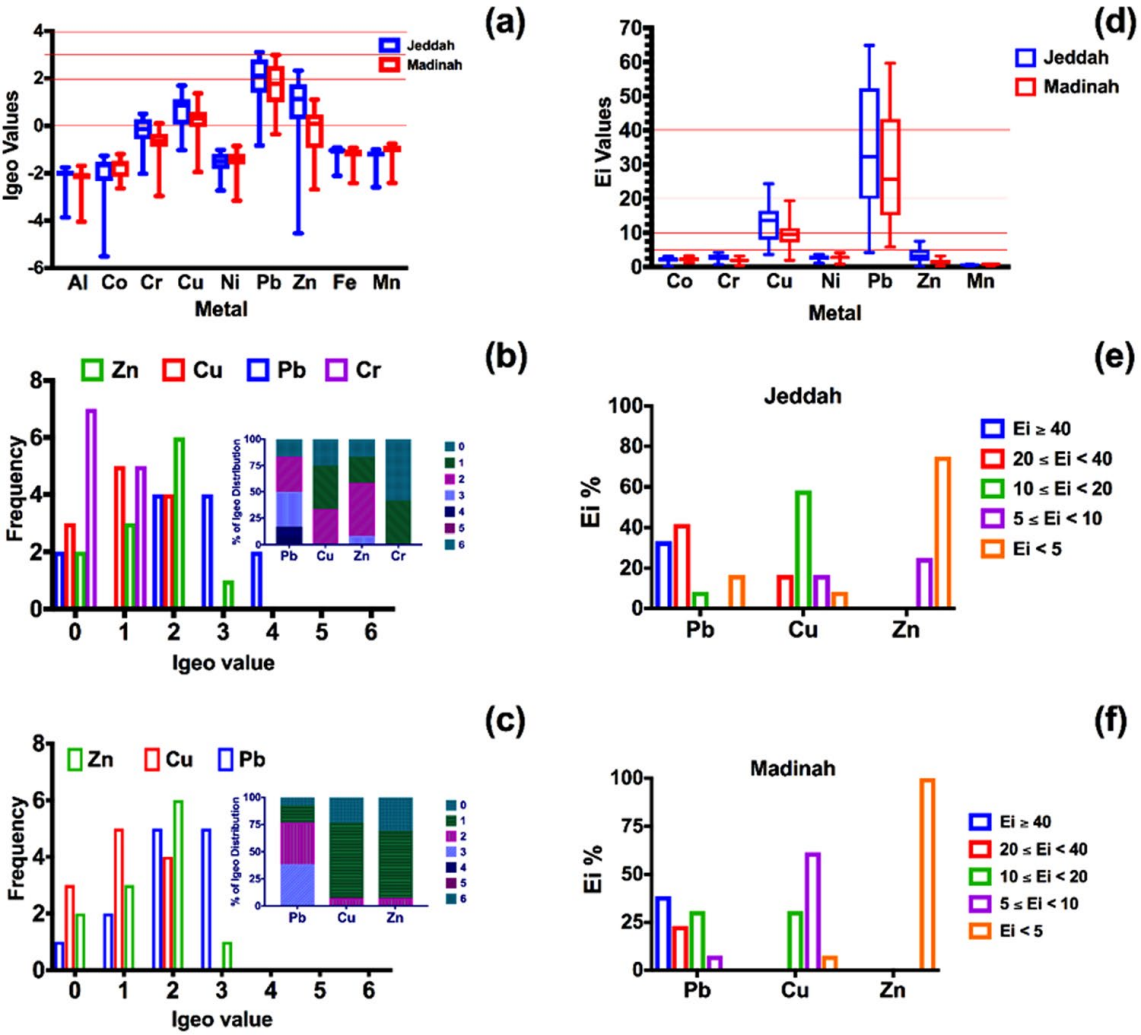
(**a**) The Box plots of the Geo-accumulation Index (Igeo-index) values of the metals associated with urban dust samples in Jeddah and Madinah (**a**). (**b**,**c**) the frequency of the Igeo-indices of the highest four metals (Zn, Cu, Pb, Cr) of the urban dust; The percentage distribution of Igeo for each metal are presented in the small graph inside the figures. (**d**) The ecological risk (E_i_) values of seven metals associated with urban dust in Jeddah and Madinah. (**e**,**f**) the percentage distribution of the most highest three metals (Pb, Cu, and Zn) in Jeddah and Madinah. First and third quartiles of the values are represented by the top and bottom of the box, respectively, the band inside the box represents the median, the error bars represent the minimum and maximum values.

The calculated Igeo values of the sampling locations of the two metropolitan cities are displayed in Fig. 4(a). Figure 4(b,c) display the frequency and percentage of the highest Igeo values in Jeddah and Madinah, respectively. The calculated Igeo values of Al, Co, Cr, Ni, Fe, and Mn in both metropolitan cities did not exceed 1, indicating no contamination of the inhabitants of the two cities was caused by these metals. The distribution of Igeo values of Pb was affected by the urban structure of the two cities. The Igeo values of the other metals were hardly related nor affected by the urban structure of the cities.

Based on the Igeo values of Pb in the two cities, two sampling locations (J3 and J10) were classified as strongly contaminated (4 > Igeo > 3). One of these locations was located near an oil refinery (J10) and the second in the city center (J3). Of the sampling location in Jeddah, 33% were classified as moderately to strongly contaminated (3 > Igeo > 2) compared to 38.5% of the sampling locations in Madinah.

Regarding Cu, 33% of the sampling locations of Jeddah were moderately contaminated (2 > Igeo > 1), and only one sampling location (M10) was moderately contaminated (2 > Igeo > 1) in Madinah. Similar to the CF and EF indices, only one sampling location was classified as moderately to strongly contaminated (3 > Igeo > 2) by Zn, namely the one near the seaport.

In Jeddah, 50% and 25% of the sampling locations were classified as moderately contaminated (2 > Igeo > 1) and uncontaminated to moderately contaminated (1 > Igeo > 0) with Zn, respectively. In Madinah, only one sampling location (M4), in the city center, was classified as moderately contaminated, and the Igeo values of the rest of the sampling locations were below 1.

In terms of comparison between the previous three indices, Supplementary Table S3 shows EF, CF, and Igeo values illustrated by three color-coded levels for selected metals (Pb, Zn, Cu, and Cr). Each color corresponds to a different broad level of contamination. These broad levels are clean to low contamination (green entries), moderate contamination (orange entries), and high contamination (red entries). Different sensitivity of indices toward contamination can be observed in Table S3. For instance, inconsistency in Zn contamination level was observed in J3, J8, J9, M1, M2, M7, M10, and M11. In each of these sampling locations, Zn contamination levels indicated by Igeo, CF, and EF were low, moderate, and high, respectively. On the other hand, the indicated classifications of some other sampling locations (J11, J12, M9, and M13) were consistent.

#### Potential ecological risk of a single metal (E_i_)

The calculated values of the potential ecological risk of a single metal in the sampling locations of the two metropolitan cities are presented in Fig. 4(d), and the classification of the ecological risk for the highest metals are shown in Fig. 4(e,f). It was observed that only three metals (Pb, Zn, and Cu) were considered to cause a moderate risk and above (Ei > 5). The highest potential risk of Pb was observed in the sampling location near the oil refinery in Jeddah (J10), followed by the sampling location in the city center (J3). In Madinah, the highest potential risk of Pb was observed in the city center (i.e., M2, M3, and M4). The potential ecological risk of Pb was related to the distribution pattern of the cities.

In Jeddah, 33% of the sampling locations were at very high ecological risk (Ei > 40) from Pb, compared to 38.5% in Madinah. Three sampling locations considered at high ecological risk (40 > Ei > 20) from Pb were observed in Jeddah (none in Madinah), and one location was considered at considerable ecological risk (20 > Ei > 10) from Pb (four in Madinah).

For Cu, high ecological risk (40 > Ei > 20) from Cu was found in only two locations in Jeddah (J2 and J4); whereas, no high ecological risk from Cu was observed for the sampling locations of Madinah. The majority of sampling locations in Jeddah and Madinah were considered at considerable ecological risk (20 > Ei > 10) from Cu, with 58.3% and 38.5%, respectively. For Zn, three sampling locations in Jeddah were considered at moderate ecological risk (10 > Ei > 5) from Zn, all of which are located near the shore of the Red Sea.

#### Nemerow single pollution index (PI_i_)

**Figure 5:**
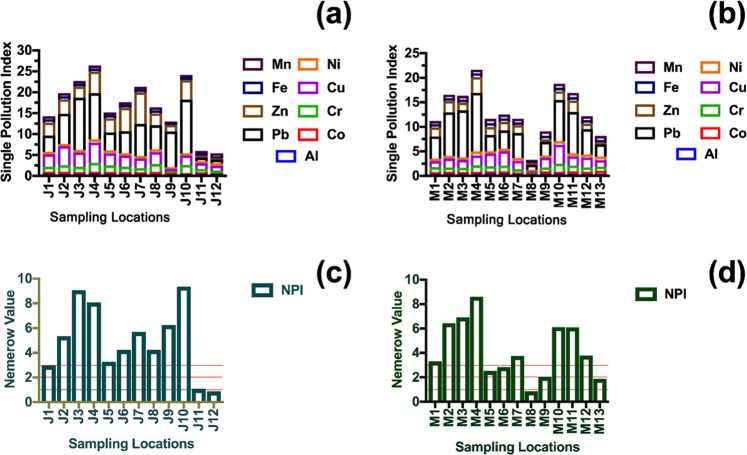
The values of Nemerow single pollution index of the metals associated with urban dust in Jeddah (**a**) and Madinah (**b**). The values of Nemerow composite pollution index of the metals associated with urban dust in Jeddah (**c**) and Madinah (**d**).

The calculated values of the single metal pollution index of the sampling locations in Jeddah and Madinah are displayed in Fig. 5(a,b). The distribution patterns of PI_i_ values of Pb and Zn were related to city structure. In Jeddah, 66% of the sampling locations were severely polluted (PI_i_ > 5) with Pb and 25% with Zn. In Madinah, almost 54% of the sampling locations were classified as severely polluted (PI_i_ > 5) with Pb, and no locations were severely polluted with Zn in Madinah. In Madinah and Jeddah, Pb was distributed in high concentrations in the city center and near the major highways. Concerning Zn in the two metropolitan cities, severely polluted (PI_i_ > 5) locations were observed only near the seaport and near the shore of the Red Sea in Jeddah.

### Contamination indices of accumulated metals

#### Nemerow Composite Pollution Index (NPI)

The calculated values of the Nemerow composite pollution index (NPI) of the sampling locations of the two metropolitan cities are presented in Fig. 5(c,d). All the investigated sampling locations in Jeddah were severely polluted (NPI > 3), except those located far from the city center and the main activities of the city (i.e., J11 and J12). Over 61% of the sampling locations in Madinah were severely polluted (NPI > 3); however, the highest NPI values were found at the sampling location near the oil refinery in Jeddah, followed by the sampling locations in the city centers of Jeddah (i.e., J3 and J4) and of Madinah (i.e., M4).

#### Pollution load index (PLI)

**Figure 6:**
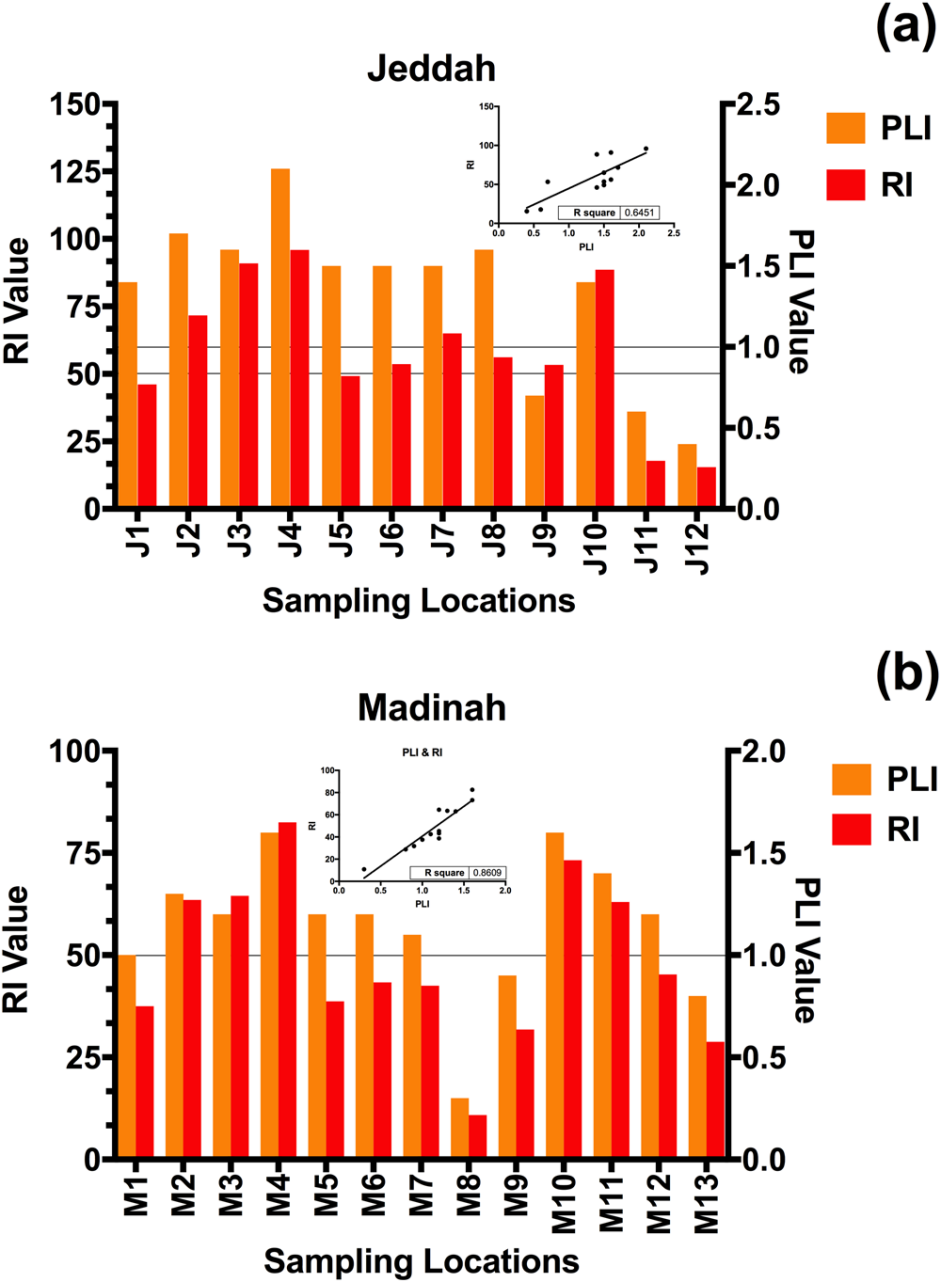
The pollution load index (PLI) and the comprehensive potential risk index (RI) values of the urban dust samples in Jeddah (**a**) and Madinah (**b**); regression lines were plotted in the upper-right space of the figures to reflect the correlation between PLI and RI.

Similar results to those of the NPI were found using the PLI index in Jeddah, as shown in Fig. 6(a), where all the sampling locations are considered deteriorated in quality (PLI > 1), except the two sampling locations located far from the city center (J11 and J12) along with the sampling location near the industrial city (J9). The sampling location of J9 was classified via the PLI as good in quality, whereas the classification via the NPI was severely contaminated, which highlights the difference in sensitivity between indices, as explained by Alharbi, *et al*.^26^. However, the distribution of PLI values was not related to the urban structure of either of the two cities.

In Madinah, more than 75% of the sampling locations had deteriorated quality (PLI > 1) as shown in Fig. 6(b). The lowest PLI values were found in the sampling locations M8, M9, and M13, which are located far from the city center (or for a site under construction and excavation, as in the case of M8).

#### Ecological risk index (RI)

The distribution of RI values better represented the structure and land use of the cities than the NPL and PLI did. However, 75% of the sampling locations in Jeddah were at high (RI > 100) and considerable (100 > RI > 50) ecological risk for the composite metals, as shown in Fig. 6(a,b). The highest ecological risk was found to be in the sampling location J4; however, these results differ from those found via the NPI index, where the highest contamination was found in the sampling locations J3 and J10.

In Madinah, 38.5% of the sampling locations were at considerable ecological risk (100 > RI > 50), as most of these locations are located in the center and along the major highways of the city.

The severity of metal contamination in Jeddah, based on the RI index, was in the following order: Pb (57.7%) > Cu (22.7%) > Zn (5.7%) > Cr (4.7%) > Ni (4.5%) > Co (3.6%) > Mn (1%). For Madinah, the order was Pb (61.2%) > Cu (20%) > Ni (5.5%) > Co (4.4%) > Cr (4%) > Zn (3.4%) > Mn (1.5%).

### Health risk assessment

#### Exposure risk analysis

**Figure 7:**
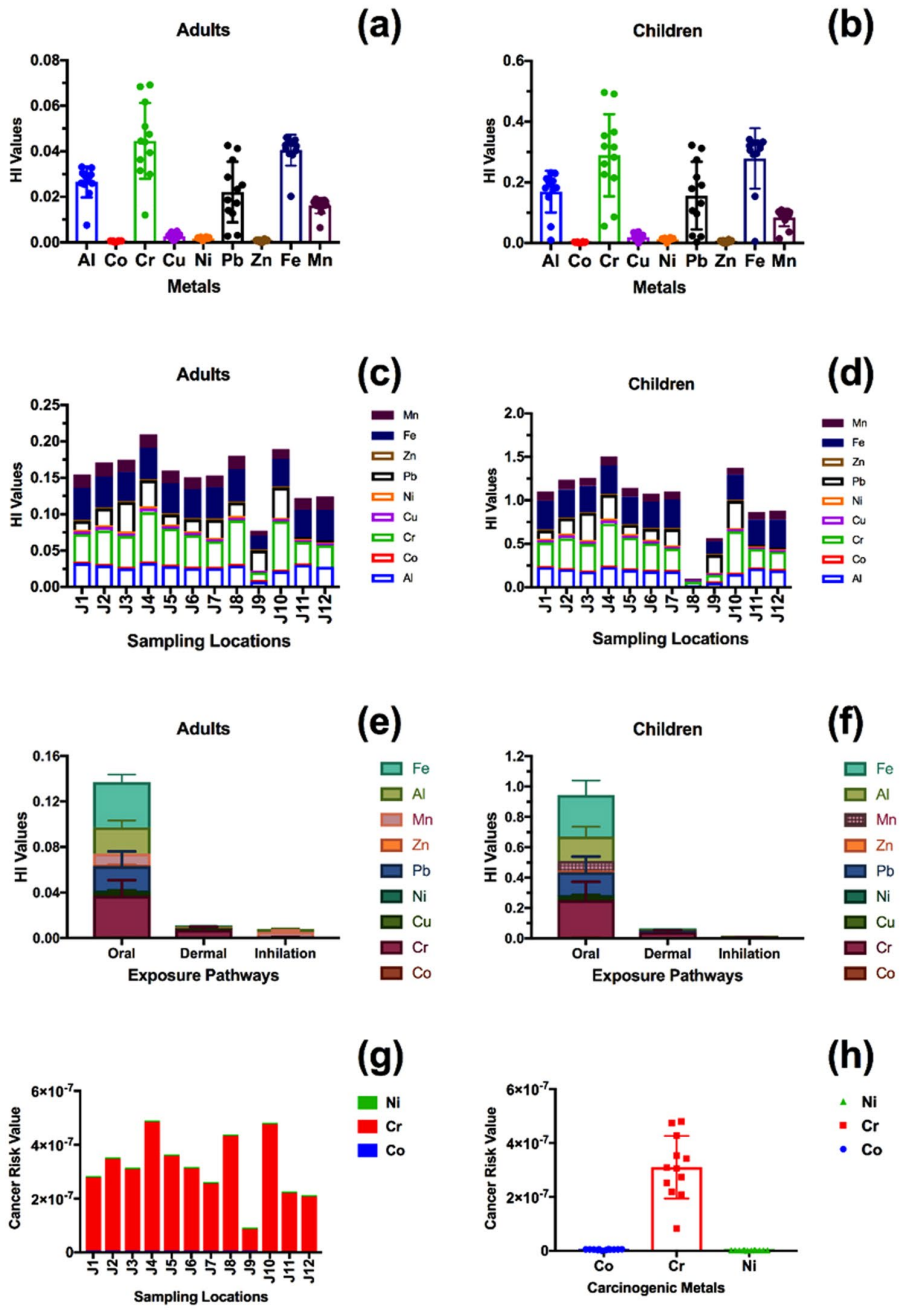
The health risk values of the metals associated with urban dust in Jeddah; (**a,b**) the average HI values of the various metals in the city, (**c,d**) the distribution of HI values in the various sampling locations, (**e,f**) the HI of the exposure pathways, (**g**) the cancer risk values in the various sampling locations, and (**h**) the average value of cancer risk.

**Figure 8:**
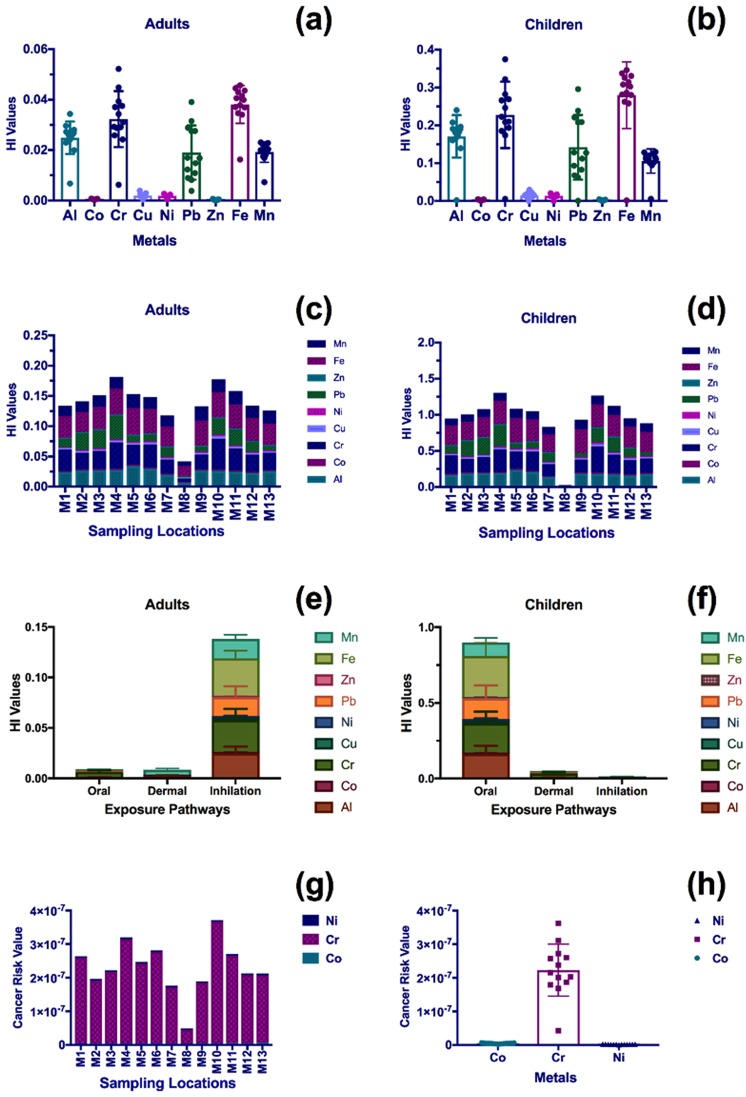
The health risk values of the metals associated with urban dust in Madinah; (**a,b**) the average HI values of the various metals in the city, (**c,d**) the distribution of HI values in the various sampling locations, (**e,f**) the HI of the exposure pathways, (**g**) the cancer risk values in the various sampling locations, and (**h**) the average value of cancer risk.

The health risk through exposure pathways to metals associated with dust for the inhabitants of the two metropolitan cities is presented in Fig. 7 for Jeddah and in Fig. 8 for Madinah. The major pathway of exposure to metals associated with urban dust in the city of Jeddah for the two age groups (i.e., adults and children) was ingestion, followed by dermal absorption and inhalation, respectively, suggesting an increased health risk related to these pathways.

In Madinah, ingestion was the major pathway of exposure to metals associated with urban dust for children, whereas inhalation was the major exposure pathway for adults.

The health risk of two routes of exposure (ingestion and dermal) to metals associated with urban dust for children was a minimum of five times higher than that of adults in the two metropolitan cities.

#### Non-cancer risk analysis

The hazard index (HI) values, representing non-carcinogenic health risk, of metals associated with urban dust in the two metropolitan cities are displayed in Figs 7 and 8. The health risk values were not related to the urban structure of the two cities. The contribution of the different metals to the non-carcinogenic risk in Jeddah for both age groups (i.e., adults and children) was in the following order: Cr > Fe > Al > Pb > Mn > Cu > Ni > Zn > Co. The contribution of metals associated with dust in Madinah was slightly different: Fe > Cr > Al > Pb > Mn > Cu > Ni > Co > Zn. However, the non-carcinogenic risk of exposure to Cr associated with urban dust was over 6 times higher for children than for adults.

Cr was a major contributor to non-carcinogenic health risk in the two metropolitan cities for the two age groups (i.e., adults and children), with a health risk contribution percentage of 29% for Jeddah and 24% for Madinah.

The highest non-carcinogenic health risk in Jeddah for the two age groups was found for the locations near the oil refinery (J10) and in the city center (J4), as shown in Fig. 7(c,d). In Madinah, the highest non-carcinogenic health risk was found in the city center (M4) and near the main highway of the city (M10), as shown in Fig. 8(c,d).

Based on the interpretation provided by the USEPA^55^, a significant health risk (non-carcinogenic risk) for children was present in 75% of the sampling locations in Jeddah and 54% in Madinah. No significant health risk (non-carcinogenic risk) existed for adults in Jeddah or Madinah.

#### Cancer risk analysis

The potential health risk of cancer caused by metals associated with urban dust was negligible in the two metropolitan cities, as shown in Figs 7(g,h) and 8(g,h); none of the HI values were higher than 1 in 1,000,000. However, Cr had the highest values of cancer risk in the two metropolitan cities, with an average value of 3.1 × 10^−7^ (±1.2 × 10^−7^) and 2.2 × 10^−7^ (±0.8 × 10^−7^) for Jeddah and Madinah, respectively.

## Conclusion

A typical pattern of compact city, where the concentrations of selected metals, namely Pb and Zn, increase toward the center, was observed in Madinah, whereas a corridor city pattern, where metal contamination was derived away from the city center, was observed in Jeddah. Various factors such as oil refineries, seaports, desalination/power plants, and industrial areas play a major role in metals accumulating in sites away from the city center, as demonstrated by the results of several contamination indices and the ecological risk index for Jeddah.

Although the different contamination indices indicate different levels of contamination at the same sampling locations, they are in relative agreement as to the severity of contamination in certain sampling locations, which might give insight to the unanimity of indices toward contamination in highly contaminated sites. For Madinah, the three composite indices of metals (NPI, PLI, and RI) were unanimous in that the sampling locations in the city center were the most contaminated; whereas for Jeddah, the most contaminated sites were those near the main activities of the city (e.g., seaport and oil refinery) and in the city center.

The potential health risk of cancer caused by metals associated with urban dust was negligible in the two metropolitan cities; however, Cr had the highest values of cancer risk in the two metropolitan cities. Cr was a major contributor of non-carcinogenic health risk in the two metropolitan cities for both adults and children, with contributions of 29% for Jeddah and 24% for Madinah. The highest non-carcinogenic health risk in Jeddah for adults and children was found for locations near the oil refinery and in the city center, whereas in Madinah, it was found in the city center and near the main highway of the city. Therefore, the distribution of health risk values showed that urban structures of the two metropolitan cities had no clear effects. Based on the findings and the limitations of this work, further research on the impact of different spatial structures on the distribution patterns of metal contamination is recommended for better urban planning and sustainability.

## Supplementary information

Supplementrary Data
