# Supplementary material for: Influence of Different Urban Structures on Metal Contamination in Two Metropolitan Cities
Source: Sci Rep. 2019 Mar 20;9:4920. doi: 10.1038/s41598-019-40180-x (PMC6426876; doi:10.1038/s41598-019-40180-x)
Supplement: Supplementary file 1 — Supplementrary Data [file 41598_2019_40180_MOESM1_ESM.docx]

**Influence of Different Urban Structures on Metal Contamination in Two Metropolitan Cities**

***Badr H. Alharbi, Mohammad J. Pasha, Mohammed Ahmad S. Al-Shamsi^*^,***

National Centre for Environmental Technology (NCET), Life Science & Environment Research Institute (LSERI), King Abdulaziz City for Science & Technology (KACST), Kingdom of Saudi Arabia

Supplementary

Figures & Tables

Table S1: Similarity Degrees of the metals in the sampling locations of the two metropolitan cities (Jeddah and Madinah) in Saudi Arabia.

Table S2: The values of the Enrichment Factors (EFs), Contamination Factors (CFs), Geo-Accumulation index (Igeo), Pollution Load Index (PLI), and Risk Index (RI) of the metals associated with the urban dust in two metropolitan cities (Jeddah and Madinah) in Saudi Arabia

Table S3: The similarities and differences in contamination levels indicated by Igeo, CF, and EF. Green entries, orange entries, and red entries represent clean to low contamination, moderate contamination, and high contamination, respectively.

| **Location** | **Cr** | | | **Cu** | | | **Pb** | | | **Zn** | | |
| --- | --- | --- | --- | --- | --- | --- | --- | --- | --- | --- | --- | --- |
|  | **EF** | **CF** | **Igeo** | **EF** | **CF** | **Igeo** | **EF** | **CF** | **Igeo** | **EF** | **CF** | **Igeo** |
| **J1** | **2.8** | **1.2** | **-0.3** | **6.8** | **3.0** | **1.0** | **8.8** | **3.9** | **1.4** | **7.1** | **3.1** | **1.1** |
| **J2** | **3.9** | **1.5** | **0.0** | **11.5** | **4.5** | **1.6** | **18.3** | **7.2** | **2.3** | **8.8** | **3.5** | **1.2** |
| **J3** | **4.0** | **1.4** | **-0.1** | **9.8** | **3.4** | **1.2** | **36.5** | **12.6** | **3.1** | **7.7** | **2.7** | **0.8** |
| **J4** | **4.8** | **2.1** | **0.5** | **10.9** | **4.9** | **1.7** | **24.7** | **11.0** | **2.9** | **11.7** | **5.2** | **1.8** |
| **J5** | **4.1** | **1.6** | **0.1** | **7.4** | **2.9** | **0.9** | **11.1** | **4.3** | **1.5** | **8.9** | **3.4** | **1.2** |
| **J6** | **3.9** | **1.4** | **-0.1** | **7.5** | **2.6** | **0.8** | **15.0** | **5.3** | **1.8** | **16.1** | **5.7** | **1.9** |
| **J7** | **3.2** | **1.1** | **-0.4** | **6.6** | **2.3** | **0.6** | **22.0** | **7.7** | **2.4** | **21.6** | **7.6** | **2.3** |
| **J8** | **4.8** | **1.9** | **0.3** | **7.1** | **2.8** | **0.9** | **14.4** | **5.7** | **1.9** | **7.1** | **2.8** | **0.9** |
| **J9** | **3.6** | **0.4** | **-2.0** | **7.2** | **0.7** | **-1.0** | **85.1** | **8.7** | **2.5** | **15.6** | **1.6** | **0.1** |
| **J10** | **7.2** | **2.1** | **0.5** | **7.9** | **2.3** | **0.6** | **44.4** | **13.0** | **3.1** | **15.9** | **4.6** | **1.6** |
| **J11** | **2.4** | **1.0** | **-0.6** | **3.4** | **1.4** | **-0.1** | **2.3** | **0.9** | **-0.7** | **0.8** | **0.3** | **-2.3** |
| **J12** | **2.5** | **0.9** | **-0.7** | **2.9** | **1.1** | **-0.5** | **2.3** | **0.8** | **-0.8** | **0.2** | **0.1** | **-4.5** |
| **M1** | **3.5** | **1.1** | **-0.4** | **3.8** | **1.2** | **-0.3** | **13.9** | **4.5** | **1.6** | **5.8** | **1.9** | **0.3** |
| **M2** | **2.3** | **0.8** | **-0.8** | **4.9** | **1.8** | **0.3** | **24.3** | **8.9** | **2.6** | **6.2** | **2.3** | **0.6** |
| **M3** | **2.6** | **1.0** | **-0.6** | **4.4** | **1.6** | **0.1** | **26.1** | **9.6** | **2.7** | **4.2** | **1.5** | **0.0** |
| **M4** | **3.7** | **1.4** | **-0.1** | **5.3** | **2.0** | **0.4** | **31.5** | **11.9** | **3.0** | **8.5** | **3.2** | **1.1** |
| **M5** | **2.3** | **1.1** | **-0.5** | **5.5** | **2.5** | **0.8** | **7.2** | **3.3** | **1.2** | **3.5** | **1.6** | **0.1** |
| **M6** | **3.0** | **1.2** | **-0.3** | **7.2** | **2.9** | **0.9** | **9.4** | **3.8** | **1.3** | **4.3** | **1.7** | **0.2** |
| **M7** | **2.8** | **0.7** | **-1.0** | **6.1** | **1.7** | **0.1** | **19.1** | **5.1** | **1.8** | **5.9** | **1.6** | **0.1** |
| **M8** | **2.1** | **0.2** | **-3.0** | **4.3** | **0.4** | **-2.0** | **12.9** | **1.2** | **-0.4** | **4.0** | **0.4** | **-2.0** |
| **M9** | **2.2** | **0.8** | **-0.9** | **5.4** | **2.0** | **0.4** | **7.5** | **2.7** | **0.9** | **1.4** | **0.5** | **-1.5** |
| **M10** | **4.6** | **1.6** | **0.1** | **11.0** | **3.9** | **1.4** | **23.7** | **8.4** | **2.5** | **5.0** | **1.8** | **0.2** |
| **M11** | **3.5** | **1.2** | **-0.4** | **5.6** | **1.9** | **0.3** | **25.1** | **8.4** | **2.5** | **7.0** | **2.3** | **0.6** |
| **M12** | **2.9** | **0.9** | **-0.7** | **6.5** | **2.0** | **0.4** | **16.7** | **5.2** | **1.8** | **3.8** | **1.2** | **-0.4** |
| **M13** | **2.6** | **0.9** | **-0.7** | **3.8** | **1.3** | **-0.2** | **7.2** | **2.5** | **0.7** | **0.7** | **0.2** | **-2.7** |
